# Impact of bladder volume and bladder shape on radiotherapy consistency and treatment interruption in prostate cancer patients

**DOI:** 10.1002/acm2.70026

**Published:** 2025-02-19

**Authors:** Sijuan Huang, Ting Li, Yujun Guo, Xiuying Mai, Xinyi Dai, Manli Wu, Mengxue He, Yang Liu, Liru He, Xin Yang

**Affiliations:** ^1^ State Key Laboratory of Oncology in South China, Guangdong Key Laboratory of Nasopharyngeal Carcinoma Diagnosis and Therapy, Guangdong Provincial Clinical Research Center for Cancer, Sun Yat‐sen University Cancer Center United Laboratory of Frontier Radiotherapy Technology of Sun Yat‐sen University & Chinese Academy of Sciences Ion Medical Technology Co., Ltd Guangzhou Guangdong P. R. China; ^2^ National Cancer Center/National Clinical Research Center for Cancer/Cancer Hospital & Shenzhen Hospital Chinese Academy of Medical Sciences and Peking Union Medical College Shenzhen China; ^3^ Department of Radiation Oncology Cancer Hospital of Shantou University Medical College Shantou P. R. China; ^4^ Department of Liver Surgery, Sun Yat‐sen University Cancer Center, State Key Laboratory of Oncology in South China, Guangdong Provincial Clinical Research Center for Cancer Sun Yat‐sen University Guangzhou Guangdong P. R. China; ^5^ Department of Radiation Oncology Anhui Wanbei Coal‐Electricity Group General Hospital Suzhou China

**Keywords:** bladder shape, bladder volume, consistency, prostate cancer, setup error, treatment interruption

## Abstract

**Background:**

To investigate the effect of bladder volume (BV) and bladder shape on consistency and treatment interruption in prostate cancer radiotherapy (RT).

**Methods:**

A total of 275 patients who underwent radical prostate cancer RT in our institution from April 2015 to December 2022 were enrolled. Bladder height, bladder width, and bladder length were defined and recorded. The receiver operating characteristic (ROC) curves were used to evaluate the best cut‐off point for bladder shape. Logistic regression analysis was used to analyze the relationship between setup errors and bladder shapes and BV.

**Results:**

Based on the ROC curves for 275 patients, the bladder shapes were classified into three: (a) the elongated bladder, (b) the spherical bladder, and (c) the oval bladder. Sixty‐six prostate cancer patients (1611 CBCTs) were randomly selected proportionally. It was found that bladder shape has a greater impact on setup errors than BV (BV: OR = 1.470, *p *= 0.037; bladder shape: OR = 2.013, *p *< 0.001), and the setup error of the spherical bladder in anterior–posterior (AP) direction was greater than the others (*p *< 0.001). In addition, the shape consistency of the spherical bladder was the worst (43.0%) during RT. Compared with the inconsistent group, the group with the same bladder shape had higher consistency in BV_(CBCT/CT)_ (*p *< 0.001), and a smaller setup error in the AP direction (*p *< 0.001). Similarly, the treatment interruption fractions were highest in spherical bladder RT.

**Conclusions:**

More specific bladder filling requirements should be developed for different bladder shapes. More attention should be paid to the spherical bladder for precise RT.

## 1 BACKGROUND

Radiotherapy (RT) is one of the radical therapies for prostate cancer and can achieve curative effects in both locally and locally advanced prostate cancer.^1^, ^2^, ^3^ With the development and popularity of intensity‐modulated radiation therapy (IMRT) and image‐guided radiation therapy (IGRT) techniques, it is possible to ensure that prostate cancer tumors receive high doses of radiation while minimizing the dose to surrounding organs at risk (OARs). Matching the Cone Beam computed tomography (CBCT) images to the planning computed tomography (pCT) images, ensures consistent tumor location and greatly reduces the toxic effects of RT.^3^, ^4^, ^5^

Despite the widespread use of IMRT and IGRT techniques, the prostate, located between the rectum and bladder, changes dramatically during radiation therapy. Variations in the location, volume, and shape of the rectum and bladder not only affect the displacement of target areas such as the prostate and seminal vesicle glands,^6^, ^7^ but also cause uncertainty in the dose to the bowel and bladder. This will increase the occurrence of acute and late gastrointestinal (GI) and acute genitourinary (GU) toxicity during RT.^8^, ^9^ Therefore, ensuring consistency of the rectum and bladder during RT is an important component of precise RT for prostate cancer. Currently, there are many studies in this area: such as the effect of the rectal and bladder alterations on tumor dosimetry and strategies of the bowel and bladder preparation.^8^, ^10^, ^11^ Nowadays, a bowel and bladder preparation process has also been summarized in our center and used in our routine clinical practice.^11^ Before daily RT, patients need to undergo bowel and bladder preparation as recommended by their physicians and monitored by CBCTs to ensure their compliance.

Many studies have focused on bladder volume (BV), such as the comparison of stability between full and empty bladders^12^ and optimal BV during RT.^13^, ^14^ However, few articles have investigated bladder shape. Several studies have shown that predictive models of bladder shape have been implemented,^15^, ^16^ but they did not analyze the differences in consistency of different bladder shapes. In our daily practice, it was found that the bowel and bladder preparations can keep BV relatively constant. However, different patients have different bladder shapes. Different bladder shapes would have different urinary BV tolerance limitations. Even if the BVs are the same, the bladder shapes can vary, potentially leading to inconsistent changes in target displacement. In addition, bladder shape can change for intra‐fraction and inter‐fraction for the same patient, potentially leading to inconsistent changes in target displacement. Therefore, the change in bladder shape was important for precise RT of prostate cancer.

This study first presented three kinds of bladder shapes and analyzed the effect of different bladder shapes for RT to provide clinical reference recommendations for the treatment of prostate cancer patients. The differences in RT consistency/setup errors among three different bladder shapes were analyzed, and the influence of bladder shape on the interruption rate of RT was also statistically analyzed.

## 2 METHODS

### 2.1 General information

A total of 275 patients with prostate cancer who received radical RT at our center from April 2015 to December 2022 were retrospectively analyzed. All patients were treated using the Elekta Versa HD (Elekta, Stockholm, Sweden) accelerator. The preparation of the bowel and bladder met the clinical requirements. The prescribed dose of the target area was 45–70 Gy, and the treatment fractions were 25–28 times. Sixty‐six patients were randomly selected from 275 patients, and daily CBCT image data were obtained. Bladder contouring of CBCT images was performed using MIM Software (Medical Image Merge, 7.1.3). A total of 1611 CBCT images with bladder contouring were obtained.

### 2.2 Bowel and bladder preparation

All prostate cancer patients underwent the bowel and bladder preparation protocol in our center.^11^ The patients underwent dietary control and bladder holding training 2 weeks prior to the immobilization to record his tolerance level of bladder filling. The patients should empty the bowel 1 h before the immobilization, CT simulation (CT‐sim), and RT. The patients had to drink 600–800 mL of water in 3–4 times after rectal emptying. We measured the BV using a BV measurement (Verathon Bladder Scan 9400), and 200–400 mL BV was considered acceptable. In addition, the rectal diameter was observed during the CT‐sim, and the rectal diameter (< 3.5 cm) at the slices of the targets (the prostate and seminal vesicle glands) was judged to be acceptable for rectal emptying. Patients who meet the requirements for bladder filling and rectal emptying will be treated, while those who do not meet the requirements will need to be repositioned or restart the bowel and bladder preparation.

### 2.3 Image acquisition and bladder contouring

CT simulating was performed on a Brilliance big bore (Philips, Amsterdam, Netherlands). Scanning parameters were as follows: voltage: 140 KV, tube current: 300 mAs, scanning slice thickness and reconstruction slice thickness: 3 mm. All images were transferred to the Monaco treatment planning system (TPS) (Version 6.01.11, Elekta). The CTV for low‐risk patients included the prostate, and for moderate‐risk patients, it included the prostate and seminal vesicles. For high‐risk and locally advanced (T3b‐T4) patients, CTV1 included the prostate and seminal vesicles, and CTV2 included the pelvic lymph nodes. CTVs were expanded by approximately 5 mm (3 mm posteriorly) to produce planning target volumes (PTVs).^17^ OARs included the rectum, bladder, femoral head, small intestine, colon, anal canal, and penis bulb, in which the rectum curved from the sigmoid colon to the bottom of ischial tuberosity (anal canal into the anus 3 cm), and the delineation of the bladder included the bladder wall. The specific constraints of targets and OARs have been shown in previous research.^17^, ^18^

The patients underwent the bowel and bladder preparation according to the doctor's advice before daily treatment. The CBCT was used for fractional positional adjustment. CBCT image acquisition parameters were as follows: voltage 120 kV, filter selected as “M,” the reconstruction resolution 410 × 410, layer thickness 3 mm, and FOV 50 cm. The CBCT images were automatically registered to the planning CT (pCT). The registration frame must include the target areas of the prostate, seminal vesicle, and pelvic lymphatic drainage. After automatic registration, appropriate manual fine‐tuning was performed according to the principle of target alignment as required by doctors. Patients who did not meet the requirements would be repositioning or restarting the bowel and bladder preparation. Finally, setup errors were recorded and collected in three dimensions: superior–inferior (SI), AP, and left–right (LR).

Both pCT and CBCT images of patients were transferred to the MIM software. The pCT was registered with the daily CBCT at the MIM, and the bladder was delineated on the CBCTs according to the patient's anatomical structure. The bladder contouring on CBCT images of all patients was done by two observers, and the BV was recorded. The ratio of CBCT/CT was used to represent the consistency of the bladder in the treatment process with that in CT‐sim, and the closer to 1 represented the higher consistency.

### 2.4 Definition of bladder diameter and three bladder shapes

**FIGURE 1:**
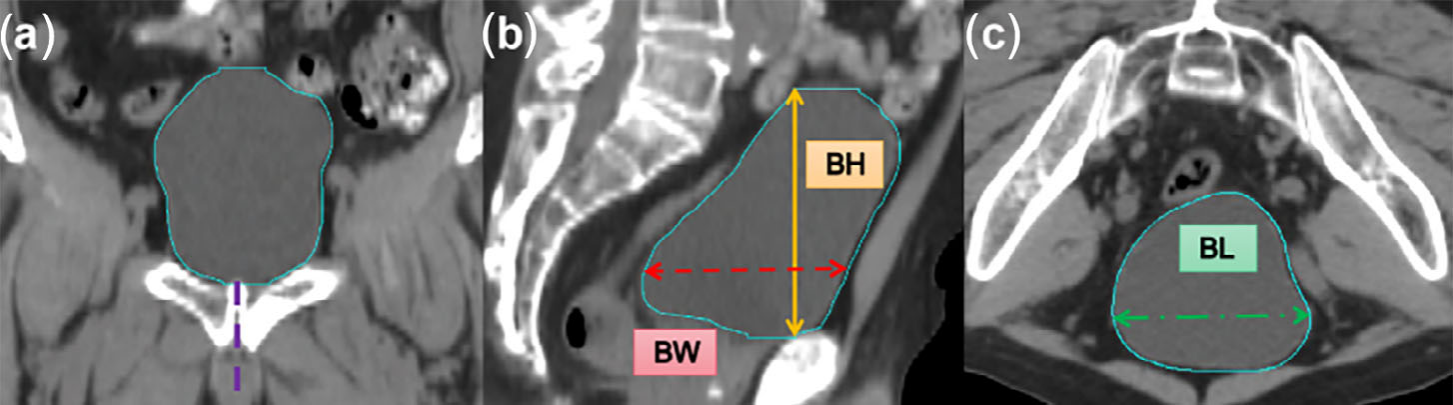
Diagram of BH, BW, and BL in the sagittal and transverse planes on CT images. (a) The purple line indicates the midline of the pubic symphysis in the CT coronal plane, which is used to determine the sagittal plane of the diameter BH. (b) The orange line represents BH. BH is the longest longitudinal diameter of the bladder in the SI direction, from the bottom to the top of the bladder. The red line represents BW, which is the longest transverse diameter of the bladder in the AP direction and is perpendicular to BH in the CT sagittal plane. (c) The green line represents the BL. BL is the longest diameter in the LR direction, located in the transverse where BW is located. BH, bladder height; BL, bladder length; BW, bladder width.

On the pCT images, the diameter of the bladder in the three‐dimensional direction was defined and recorded as bladder height (BH), bladder width (BW), and bladder length (BL), respectively. BH is the longest longitudinal diameter in the SI direction and was defined from the base to the top of the bladder at the sagittal in the midline of the pubic symphysis. BW is the longest transverse diameter in the AP direction, which was perpendicular to BH in the sagittal. BL has the longest diameter in the LR direction, located in the transverse where BW is located (Figure 1). The receiver operating characteristic (ROC) curve was used to quantitatively distinguish BH/BW to evaluate the best cut‐off point for bladder shape.

### 2.5 Statistical analysis

All data in the study were displayed as mean ± SD. SPSS 25.0 statistical software was used for data analysis, and *p* < 0.05 was considered to have significant statistical difference. The Kolmogorov–Smirnov test was used to test the normality of the data. One‐way ANOVA was used to compare BV and diameters under different bladder shapes if the data conformed to normal distribution; otherwise, the Kruskal–Wallis test was used. The Mann–Whitney *U* test was used to compare the consistency and inconsistency of bladder shape during RT.

## 3 RESULTS

### 3.1 Classification of the three bladder shapes

**FIGURE 2:**
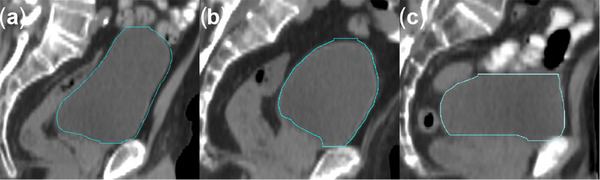
Diagram of three bladder shapes on CT sagittal plane. (a) The elongated bladder (*N* = 120): BH/BW ≥ 1.036. (b) The spherical bladder (*N* = 86): 0.836 < BH/BW < 1.036. (c) The oval bladder (*N* = 69): BH/BW ≤ 0.836. BH, bladder height; BW, bladder width.

The median age of the 275 patients was 73 years (range: 37–93 years). The BH/BW on 275 pCTs was analyzed to determine the optimal thresholds for the bladder shapes by the ROC curve. The optimal BH/BW cut‐off determined by ROC curves and the Youden test were: cut‐off value‐1 = 1.036 (AUC = 0.868; *p *< 0.001; 95% CI = 0.823–0.911) and cut‐off value‐2 = 0.836 (AUC = 0.973; *p *< 0.001; 95% CI = 0.953–0.993). Therefore, the bladder shape was classified into three types: (a) the elongated bladder (*N* = 120): BH/BW value ≥ 1.036, (b) the spherical bladder (*N* = 86): BH/BW value between 0.836 and 1.036, and (c) the oval bladder (*N* = 69): BH/BW value ≤ 0.836 (Figure 2).

Except for BL, there was a statistical difference in BV and diameters among the three bladder shapes (*p *< 0.05). The BV of the elongated bladder, the spherical bladder, and the oval bladder were 398.39 ± 102.86, 367.67 ± 114.82, and 325.51 ± 103.75 mL, respectively (Table 1).

**TABLE 1:** Comparison of BV and diameters of three bladder shapes in 275 patients.

	Elongated	Spherical	Oval	P1	P2	P3
	Bladder	Bladder	Bladder			
	(*N* = 120)	(*N* = 86)	(*N* = 69)			
BV and diameters (CT)						
BV_(mL)_	398.39 ± 102.86	367.67 ± 114.82	325.51 ± 103.75	**0.043**	**< 0.001**	**0.015**
BH_(cm)_	9.82 ± 1.21	8.28 ± 1.09	6.82 ± 1.10	**< 0.001**	**< 0.001**	**< 0.001**
BW_(cm)_	8.03 ± 1.00	8.89 ± 1.16	9.70 ± 1.25	**< 0.001**	**< 0.001**	**< 0.001**
BL_(cm)_	8.32 ± 0.99	8.17 ± 0.93	8.15 ± 1.04	0.303	0.273	0.900
BH/BW	1.23 ± 0.18	0.93 ± 0.06	0.71 ± 0.10	**< 0.001**	**< 0.001**	**< 0.001**

Abbreviations: BH, bladder height; BL, bladder length; BV, bladder volume; BW, bladder width; P1: the elongated bladder versus the spherical bladder, P2: the elongated bladder versus the oval bladder, P3: the spherical bladder versus the oval bladder.

*p *< 0.05 indicates significant difference.

### 3.2 Effects of bladder shapes and BV changes on the setup error of RT

We analyzed the changes in the three bladder shapes and the BV during treatment based on 66 pCT, 1611 CBCT images, and registration setup errors. The setup errors in the three directions were divided into two groups: < 0.5 cm and ≥ 0.5 cm.

**FIGURE 3:**
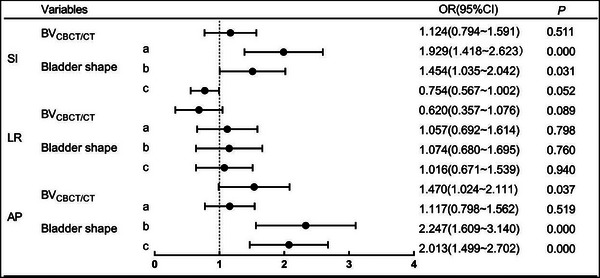
Independent predictors of large setup errors in multivariate logistic regression analysis. (The setup errors were divided into two groups: ≥ 0.5 cm and < 0.5 cm. Solid circles represented OR values, and the length of the line next to the circle represented the 95% confidence interval. OR values < 1 indicated that the variable was a protective factor, while OR > 1 indicated that the variable was a risk factor. (a) The elongated bladder versus the oval bladder; (b) the spherical bladder versus the oval bladder; (c) the elongated bladder versus the spherical bladder. Bold indicates the significant difference (*p *< 0.05).

Figure 3 summarizes the relationship of setup errors (< 0.5 cm and ≥ 0.5 cm) and bladder parameters. On multivariate analysis, it was demonstrated that bladder shape and BV are independent influencing factors of setup errors (*p *< 0.05). There are differences in setup errors and bladder parameters in the AP direction, and the bladder shape has a greater influence on the setup errors than the BV (BV: OR = 1.470, *p *= 0.037; bladder shape: OR = 2.013, *p *< 0.001).

#### 3.2.1 The influence of bladder shape on setup errors

When the bladder was the same volume, compared to patients with an oval bladder, patients with spherical bladder and elongated bladder were more likely to develop SI setup errors ≥ 0.5 cm, respectively (spherical bladder: OR = 1.454; *p *= 0.031; 95% CI = 1.035–2.042; elongated bladder: OR = 1.929; *p *< 0.001; 95% CI = 1.418–2.623). Similarly, compared to patients with spherical bladder, patients with elongated bladder and oval bladder were more likely to develop AP setup errors < 0.5 cm, respectively (oval bladder: OR = 2.247; *p *< 0.001; 95% CI = 1.609–3.140, elongated bladder: OR = 2.013; *p *< 0.001; 95% CI = 1.499–2.702). Overall, the setup errors of the oval bladder in the SI direction were the smallest, the spherical bladder in the AP direction was the largest, and there was no significant difference in the three bladder shapes in the LR direction.

Three different shapes of three‐dimensional setup errors were compared. In the SI direction, there was a statistical difference in the setup errors of the elongated bladder(0.33 ± 0.29 cm) and the oval bladder (0.27 ± 0.19 cm) (*p *= 0.030). There was a significant difference in the setup errors of different bladder shapes in the AP direction. The setup errors in the AP direction of the elongated bladder (0.26 ± 0.25 cm) and the oval bladder (0.24 ± 0.26 cm) were lower than that of the spherical bladder (0.35 ± 0.30 cm) (*p *< 0.001). There was no statistical difference between the elongated bladder and the oval bladder (*p = *0.103) (Table S1). Comparing setup errors of the three bladder shapes, the results show that the spherical bladder has the highest AP setup error.

If the bladder shape during RT was consistent with that of the pCT, it was named the consistent group; otherwise, it was named the inconsistency group. The overall consistency of the three bladder shapes was 61.1%. The BV_CBCT/CT_, BH_CBCT/CT_, and BL_CBCT/CT_ of the bladder consistency group were superior to those of the inconsistency group, with respective values of 0.78 ± 0.33, 0.91 ± 0.18, and 0.9 ± 0.13 (*p* < 0.05), respectively. There was no significant difference in BW_CBCT/CT_ between the two groups. In addition, the setup error of the AP direction with a consistent shape was smaller than that of the inconsistency group, and there was no statistical difference between the SI and LR directions. Details can be found in Table S2.

**FIGURE 4:**
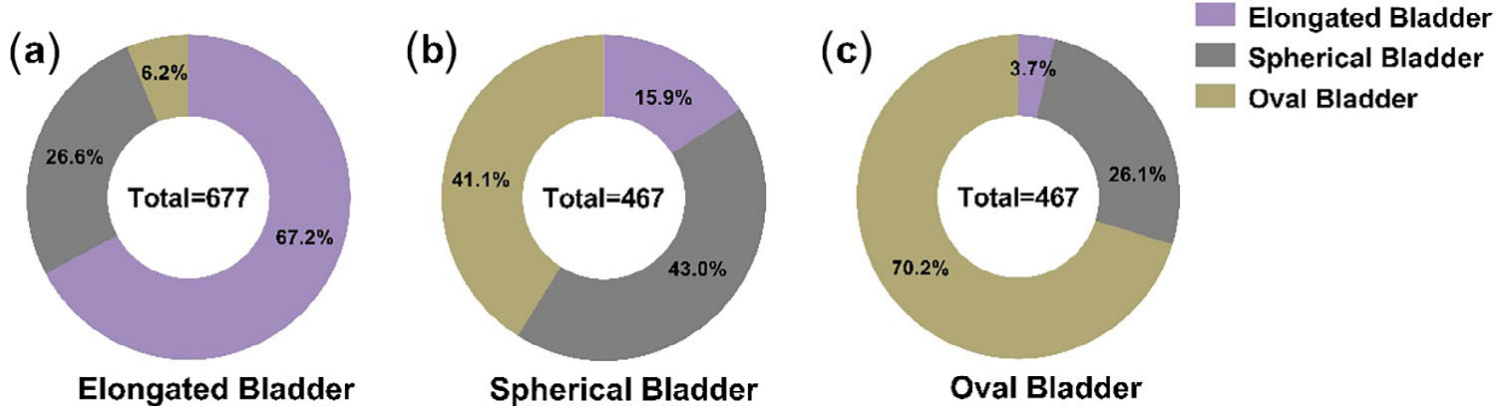
The percentage of distribution of shape consistency across three bladder shapes during treatment (classification of bladder shapes according to CT images: (a) the elongated bladder, (b) the spherical bladder, (c) the oval bladder).

As Figure 4 showed, the oval bladder had the highest shape consistency (70.2%), followed by the elongated bladder (67.2%), and the spherical bladder had the lowest shape consistency (43.0%). From the perspective of consistency in bladder shape during treatment, oval bladder was the best, followed by elongated bladder, and spherical bladder was the worst. More than half of the data on spherical bladder shapes changed during RT, and we can see from Table S2 that this change will lead to changes in BV and diameter.

#### 3.2.2 The influence of BV on setup errors

When the bladder was the same shape, patients with increased BV_CBCT/CT_ were more likely to develop AP setup errors ≥ 0.5 cm (OR = 1.470; *p *= 0.037; 95% CI = 1.024–2.111). The optimal BV_CBCT/CT_ cut‐off determined by ROC curves and the Youden test were cut‐off = 0.580 (AUC = 0.550; *p *= 0.007; 95%CI = 0.515–0.586). Therefore, patients with BV_CBCT/CT_ ≥ 58 % had higher rates of AP setup errors ≥ 0.5 cm than patients with BV < 58% (20.3%, 219/1081 vs. 13.8%, 73/530, *p *< 0.001).

### 3.3 The percentage of treatment interruption of different bladder shapes during RT

Treatment records of 66 prostate cancer patients were analyzed, and 25 patients got repeated IGRT in 1‐day due to target mismatches. Some patients were treated for the day after repeated scanning, but some patients had to interrupt the RT. After dividing the bladder shape into three groups, we found that compared to the elongated bladder (2.22%) and the oval bladder (3.21%), the interruption of the spherical bladder treatment is larger (6.00%) (Table 2).

**TABLE 2:** The percentage of distribution of shape interruption in three bladder shapes during treatment.

Bladder shape	Amount of repeated scanning	Interruption fraction	Amount of patients interrupted	Patients amount	Total fractions
Elongated bladder	28(4.14%)	15(2.22%)	6	28	677
Spherical bladder	37(7.92%)	28(6.00%)	7	19	467
Oval bladder	20(4.28%)	15(3.21%)	5	19	467

## 4 DISCUSSION

This study, as far as we know, is the first one to study the bladder shapes during RT. We found that there are three common bladder shapes: the elongated bladder, the spherical bladder, and the oval bladder. Based on our statistical results and clinical experience, the most common is the elongated bladder, followed by the spherical bladder and oval bladder. By analyzing the ROC curves of BH/BW, the optimal cut‐off values‐1 (1.036) and cut‐off value‐2 (0.836) are obtained, and the three bladder shapes are distinguished based on the cut‐off values, which was consistent with the subjective classification of RT doctors.

There are a few publications^19^, ^20^, ^21^ that discuss the relationship between BV and setup errors or prostate motion; however, there is little research on the impact of bladder shape on RT. Our study is the first to investigate the influence of bladder shape on setup errors, bladder consistency, and treatment interruption in prostate cancer during RT. By studying 275 prostate cancer patients who underwent qualified bowel and bladder preparation, the BV on pCT for the three bladder shapes were: the elongated bladder (398.39 ± 102.86 mL), the spherical bladder (367.67 ± 114.82 mL) and the oval bladder (325.51 ± 103.75 mL). All three types of BVs are similar to the requirements of the current articles reporting on the filling of BV in prostate RT,^13^, ^14^, ^22^ indicating the effectiveness of the bowel and bladder preparation before RT in our center. Statistically significant differences can be found in BV and diameters in the three bladder shapes (Table 1), indicating the effectiveness of bladder shape classification and the differences in the ability of different bladder shapes to hold urine. It was shown that the elongated bladder tolerates more urine than the Spherical Bladder and the oval bladder, and the oval bladder tolerates the least. It helps the radiation physicians or therapists judge whether the urine volume was appropriate based on the bladder shape of the patient, instead of the idea that the more urine, the better. The results that patients with different bladder shapes should have urine would reduce urinary incontinence during RT, providing more refined guidance for bladder preparation and improving the existing workflow. More specific bladder filling requirements should be proposed for different bladder shapes in subsequent clinical workflow.

To further explore the influencing factors of bladder shape, we divided age into three groups (a) 37–69, (b) 70–77, (c) 78–93. Correlation analysis was conducted between age groups and BV_CBCT/CT_, as well as bladder shape and setup errors. It was found that only BV_CBCT/CT_ and setup errors in the LR direction were correlated with age groups (*p *< 0.001), with correlation coefficients *R* of −0.227 and 0.161, respectively. There were significant differences in BV_CBCT/CT_ among the patients of the three age groups, and the oldest group were the least stable BV_CBCT/CT_, while group a had the best stability. Moreover, the LR setup error of group c was the largest (a: 0.18 ± 0.16 cm, b: 0.17 ± 0.16 cm, c: 0.25 ± 0.2 cm; *p *< 0.001). The analysis of bladder shape using the chi‐square test showed that different age groups had no affect on the bladder shape of patients (*p* = 0.338). In summary, bladder shape was not related to age, but age can affect the consistency of BV and increase setup errors in the LR direction. Therefore, more attention should be given to elderly patients.

Previous research has found that the BV tends toward significance as a factor acting on prostate motion in the AP direction.^23^ In this retrospective study, we evaluated the impact of BV and bladder shape on setup errors and found that bladder shape has a greater impact on AP direction setting errors than BV. Through multivariate logistic regression analysis, it was found that the spherical bladder is more prone to setup errors > 0.5 cm in the AP direction than the others. To demonstrate this, we analyzed the setup errors in the three directions of the three types of bladder shape. It was found that less setup error in the AP direction for the oval bladder and the elongated bladder than that for the spherical bladder (*p* < 0.05) (Table S1). Therefore, when the patient's bladder shape is found to be elongated after scanning pCT, special attention should be paid to the position and registration of the AP direction during the subsequent treatment process.

For the reproducibility of the bladder shape, Heidi T. Lotz et al.^16^ compared bladder shapes of equal volume, and found that the cranial and posterior parts of the bladder were much less reproducible. In our study, we found that all three bladder shapes would be changed during the treatment course. The oval bladder had the highest shape consistency of 70.2%, followed by the elongated bladder of 67.2% and the spherical bladder had the lowest shape consistency of 43.0%. We found that 41.1% of spherical bladders changed into the oval bladder during RT, which may be related to the fact that the spherical bladder did not achieve optimal BV during localization and CT simulation, resulting in a more variable bladder shape during later treatment (Figure 4). In terms of RT repeatability, the spherical bladder had the worst RT repeatability. This means that based on the existing clinical protocol for patients with spherical bladder, RT doctors and therapists should pay more attention to patients with spherical bladder and perform adaptive RT if necessary. In addition, the setup errors of the group with consistent bladder shape in the AP direction are smaller than those of the inconsistent group (*p *< 0.001) (Table S2). During the RT process, the bladder shape remains consistent, and the setup error is smaller. It is proposed that specific bladder preparation requirements should be made based on different bladder shapes to keep consistency of bladder shape and less setup errors during the RT course.

We collected treatment records from patients and found that 18 patients appeared RT interruptions throughout the course of RT, meaning they received CBCT scans on the same day but did not receive treatment. It was found that the spherical bladder had the highest interruption rate (6.00%). In summary, the AP direction setup errors of the spherical bladder are large, the bladder shape consistency is the worst, and the RT interruption rate is high. Therefore, more attention should be paid to the spherical bladder during clinical treatment.

For prostate cancer RT, daily IGRT is widely recognized.^24^ In clinical practice, if the target area is shifted due to the filling status of the bowel and bladder, or if more OARs are overexposed, patients have to re‐start the bowel and bladder preparation. If more preparations fail to meet the requirements, treatment will be suspended on the day. Suspended treatment to some extent can affect the efficacy of RT, so we analyzed the re‐IGRT or interruption of different bladder shapes in RT. The results showed that the interruption frequency of the spherical bladder was significantly higher than that of the elongated bladder and oval bladder. On the other hand, it also confirms that the consistency of the spherical bladder is worse than the other two bladder shapes. It further suggested the necessity for more attention to patients with spherical bladders during the RT. Additionally, bladder shapes should be considered as a key trigger condition for adaptive RT in pelvic tumor treatments.

The present study has some limitations. First, the sample size varied among the three bladder shapes collected. In addition, the poor quality of some CBCT images may affect the accurate outline of bladder structures to some extent. Despite these limitations, the literature reports for the first time about the differences in setup errors, bladder consistency, and treatment interruption rate under different bladder shapes in RT for prostate cancer. Therefore, we hope that the present results will provide more clinical guidance for precise RT. Furthermore, future studies would include the dose changes caused by shape changes during RT and the effect of different bladder shapes on the dose in the target area of prostate cancer.

## 5 CONCLUSIONS

The bladder shape has a greater impact on setup errors compared to BV, so in clinical practice, we should pay more attention to changes in bladder shape. In the future, RT fixation, CT‐sim scanning and treatment need to pay more attention to the bladder shape, especially the spherical bladder. The spherical bladder has the worst consistency and setup error during treatment, and it has the highest treatment interruption of RT. Therefore, stricter and closer attention is needed in the future. And in subsequent clinical examinations, more specific bladder filling requirements should be developed for different bladder shapes.

## AUTHOR CONTRIBUTIONS

*Conception and design*: Sijuan Huang and Liru He. *Administrative support*: Xin Yang. *Provision of study materials or patients*: All authors. *Collection and assembly of data*: All authors. *Data analysis and interpretation*: Ting Li and Yujun Guo. *Manuscript writing*: Sijuan Huang, Ting Li, Xiuying Mai, and Xin Yang. *Final approval of manuscript*: All authors.

## CONFLICT OF INTEREST STATEMENT

The authors declare no conflicts of interest.

## ETHICS STATEMENT

Our study was approved by the Institutional Ethics Committee (No: B2020‐074‐01).

## Supporting information



Table S1 Comparison of BV and diameters consistency and setup errors for three bladder shapes during treatment

Table S2 Comparison of consistency and inconsistency of bladder shape during treatment

## Data Availability

The materials and clinical data were fully available in the Research Data Deposit (RDD) Public Platform (No.: 2022914178, www.researchdata.org.cn).

## References

[1] Evans AJ . Treatment effects in prostate cancer. Mod Pathol. 2018;31(1):S110‐S121. doi:10.1038/modpathol.2017.158 29297495

[2] Valle LF , Lehrer EJ , Markovic D , et al. A systematic review and meta‐analysis of local salvage therapies after radiotherapy for prostate cancer (MASTER). Eur Urol. 2021;80(3):280‐292. doi:10.1016/j.eururo.2020.11.010 33309278 PMC10262981

[3] Bauman G , Rumble RB , Chen J , Loblaw A , Warde P , Members of the IMRT Indications Expert Panel . Intensity‐modulated radiotherapy in the treatment of prostate cancer. Clin Oncol (R Coll Radiol). 2012;24(7):461‐473. doi:10.1016/j.clon.2012.05.002 22673744

[4] Osman SOS , Russell E , King RB , et al. Fiducial markers visibility and artefacts in prostate cancer radiotherapy multi‐modality imaging. Radiat Oncol. 2019;14(1):237. doi:10.1186/s13014-019-1447-1 31878967 PMC6933910

[5] Zelefsky MJ , Kollmeier M , Cox B , et al. Improved clinical outcomes with high‐dose image guided radiotherapy compared with non‐IGRT for the treatment of clinically localized prostate cancer. Int J Radiat Oncol Biol Phys. 2012;84(1):125‐129. doi:10.1016/j.ijrobp.2011.11.047 22330997

[6] Pearson D , Gill SK , Campbell N , et al. Dosimetric and volumetric changes in the rectum and bladder in patients receiving CBCT‐guided prostate IMRT: analysis based on daily CBCT dose calculation. J Appl Clin Med Phys. 2016;17(6):107‐117. doi:10.1120/jacmp.v17i6.6207. Published 2016 Nov 8. 27929486 PMC5690499

[7] Ma TM , Neylon J , Casado M , et al. Dosimetric impact of interfraction prostate and seminal vesicle volume changes and rotation: a post‐hoc analysis of a phase III randomized trial of MRI‐guided versus CT‐guided stereotactic body radiotherapy. Radiother Oncol. 2022;167:203‐210. doi:10.1016/j.radonc.2021.12.037 34979212

[8] Chen Z , Yang Z , Wang J , Hu W . Dosimetric impact of different bladder and rectum filling during prostate cancer radiotherapy. Radiat Oncol. 2016;11:103. doi:10.1186/s13014-016-0681-z 27485637 PMC4969718

[9] Achard V , Zilli T . Prostate cancer intensity‐modulated radiotherapy and long term genitourinary toxicity: an evolving therapeutic landscape. Prostate Cancer Prostatic Dis. 2023;26(1):1‐2. doi:10.1038/s41391-022-00535-4 35488121

[10] Nightingale H , Conroy R , Elliott T , Coyle C , Wylie JP , Choudhury A . A national survey of current practices of preparation and management of radical prostate radiotherapy patients during treatment. Radiography (Lond). 2017;23(2):87‐93. doi:10.1016/j.radi.2017.01.003 28390554

[11] Huang S , Zhong Z , Pang Y , et al. Validation of bowel and bladder preparation by rectum and bladder variation in prostate radiotherapy based on cone beam CTs. J Radiat Res Appl Sci. 2023;16:100513.

[12] Pinkawa M , Asadpour B , Gagel B , Piroth MD , Holy R , Eble MJ . Prostate position variability and dose‐volume histograms in radiotherapy for prostate cancer with full and empty bladder. Int J Radiat Oncol Biol Phys. 2006;64(3):856‐861. doi:10.1016/j.ijrobp.2005.08.016 16243443

[13] Chen HH , Lin PT , Kuo LT , Lin KS , Fang CC , Chi CC . Bladder volume reproducibility after water consumption in patients with prostate cancer undergoing radiotherapy: a systematic review and meta‐analysis. Biomed J. 2021;44(6) (suppl 2):S226‐S234. doi:10.1016/j.bj.2020.11.004 35300945 PMC9068550

[14] Fujioka C , Ishii K , Yamanaga T , et al. Optimal bladder volume at treatment planning for prostate cancer patients receiving volumetric modulated arc therapy. Pract Radiat Oncol. 2016;6(6):395‐401. doi:10.1016/j.prro.2016.05.007 27374192

[15] Lotz HT , Remeijer P , van Herk M , et al. A model to predict bladder shapes from changes in bladder and rectal filling. Med Phys. 2004;31(6):1415‐1423. doi:10.1118/1.1738961 15259644

[16] Lotz HT , van Herk M , Betgen A , Pos F , Lebesque JV , Remeijer P . Reproducibility of the bladder shape and bladder shape changes during filling. Med Phys. 2005;32(8):2590‐2597. doi:10.1118/1.1992207 16193789

[17] Liu YP , Gray PJ , Jin J , et al. Hypofractionated intensity‐modulated radiation therapy for prostate cancer confined to the pelvis: analysis of efficacy and late toxicity. J Radiat Oncol. 2015;4:95‐101.

[18] Zhou S , Luo L , Li J , et al. Analyses of the factors influencing the accuracy of three‐dimensional ultrasound in comparison with cone‐beam CT in image‐guided radiotherapy for prostate cancer with or without pelvic lymph node irradiation. Radiat Oncol. 2019;14(1):22. doi:10.1186/s13014-019-1217-0 30696488 PMC6352439

[19] Meijer GJ , Rasch C , Remeijer P , Lebesque JV . Three‐dimensional analysis of delineation errors, setup errors, and organ motion during radiotherapy of bladder cancer. Int J Radiat Oncol Biol Phys. 2003;55(5):1277‐1287. doi:10.1016/s0360-3016(02)04162-7 12654438

[20] Kristiansen NK , Ringgaard S , Nygaard H , Djurhuus JC . MRI assessment of the influence of body position on the shape and position of the urinary bladder. Scand J Urol Nephrol. 2004;38(1):53‐61. doi:10.1080/00365590310017325 15204428

[21] Roch M , Zapatero A , Castro P , Hernández D , Chevalier M , García‐Vicente F . Dosimetric impact of rectum and bladder anatomy and intrafractional prostate motion on hypofractionated prostate radiation therapy. Clin Transl Oncol. 2021;23(11):2293‐2301. doi:10.1007/s12094-021-02628-3 33913091

[22] Mullaney LM , O'Shea E , Dunne MT , et al. A randomized trial comparing bladder volume consistency during fractionated prostate radiation therapy. Pract Radiat Oncol. 2014;4(5):e203‐e212. doi:10.1016/j.prro.2013.11.006 25194106

[23] Marnouche EA , Hadadi K , Abdelhak M , et al. Evaluation of margins in pelvic lymph nodes and prostate radiotherapy and the impact of bladder and rectum on prostate position. Cancer Radiother. 2021;25(2):161‐168. doi:10.1016/j.canrad.2020.06.033 33454191

[24] di Franco F , Baudier T , Pialat PM , et al. Ultra‐hypofractionated prostate cancer radiotherapy: dosimetric impact of real‐time intrafraction prostate motion and daily anatomical changes. Phys Med. 2024;118:103207. doi:10.1016/j.ejmp.2024.103207 38215607

