# Supplementary material for: Impact of bladder volume and bladder shape on radiotherapy consistency and treatment interruption in prostate cancer patients
Source: J Appl Clin Med Phys. 2025 Feb 19;26(4):e70026. doi: 10.1002/acm2.70026 (PMC11969095; doi:10.1002/acm2.70026)
Supplement: Supplementary file 1 — Table S1 Comparison of BV and diameters consistency and setup errors for three bladder shapes during treatment [file ACM2-26-e70026-s001.docx]

**Table S1 Comparison of BV and diameters consistency and setup errors for three bladder shapes during treatment**

|  | Elongated | Spherical | Oval | *P1* | *P2* | *P3* |
| --- | --- | --- | --- | --- | --- | --- |
|  | Bladder | Bladder | Bladder |  |  |  |
|  | N=(28) | N=(19) | N=(19) |  |  |  |
| BV and diameters (CT) |  |  |  |  |  |  |
| BV_(ml)_ | 362.17±107.73 | 354.26±112.27 | 346.2±103.96 | 0.455 | **0.002** | **0.030** |
| BH_(cm)_ | 9.49±1.24 | 8.12±1.01 | 6.96±1.12 | **<0.001** | **<0.001** | **<0.001** |
| BW_(cm)_ | 7.62±0.89 | 8.65±0.97 | 9.98±1.22 | **<0.001** | **<0.001** | **<0.001** |
| BL_(cm)_ | 7.95±0.99 | 8.05±0.93 | 8.57±0.80 | **0.007** | **<0.001** | **<0.001** |
| BH/BW | 1.25±0.13 | 0.94±0.06 | 0.71±0.12 | **<0.001** | **<0.001** | **<0.001** |
| BV and diameters (CBCT/CT) |  |  |  |  |  |  |
| BV | 0.77±0.36 | 0.72±0.29 | 0.75±0.37 | 0.190 | 0.300 | 0.867 |
| BH | 0.87±0.20 | 0.85±0.20 | 0.93±0.22 | 0.120 | **<0.001** | **<0.001** |
| BW | 0.95±0.18 | 0.91±0.12 | 0.85±0.13 | **<0.001** | **<0.001** | **<0.001** |
| BL | 0.91±0.15 | 0.89±0.11 | 0.88±0.12 | **0.042** | **<0.001** | 0.069 |
| Setup error (cm) |  |  |  |  |  |  |
| SI | 0.33±0.29 | 0.29±0.23 | 0.27±0.19 | 0.228 | **0.030** | 0.328 |
| LR | 0.20±0.17 | 0.22±0.19 | 0.20±0.18 | 0.198 | 0.713 | 0.124 |
| AP | 0.26±0.25 | 0.35±0.30 | 0.24±0.26 | **<0.001** | 0.103 | **<0.001** |
| Total fractions | 677 | 467 | 467 |  |  |  |

*P1*: the Elongated Bladder vs the Spherical Bladder, *P2*: the Elongated Bladder vs the Oval Bladder, *P3*: the Spherical Bladder vs the Oval Bladder, Bold indicates the significant difference (*P*<0.05).
