# Supplementary material for: Impact of bladder volume and bladder shape on radiotherapy consistency and treatment interruption in prostate cancer patients
Source: J Appl Clin Med Phys. 2025 Feb 19;26(4):e70026. doi: 10.1002/acm2.70026 (PMC11969095; doi:10.1002/acm2.70026)
Supplement: Supplementary file 2 — Table S2 Comparison of consistency and inconsistency of bladder shape during treatment [file ACM2-26-e70026-s002.docx]

**Table S2 Comparison of consistency and inconsistency of bladder shape during treatment**

| bladder shape | Consistency of bladder shape | BV_CBCT/CT_ | BH_CBCT/CT_ | BW_CBCT/CT_ | BL_CBCT/CT_ | SI(cm) | LR(cm) | AP(cm) | N |
| --- | --- | --- | --- | --- | --- | --- | --- | --- | --- |
| Elongated 28 | Consistency | 0.84±0.37 | 0.93±0.18 | 0.94±0.18 | 0.93±0.15 | 0.33±0.30 | 0.20±0.16 | 0.25±0.25 | 455(67.2%) |
|  | Inconsistency | 0.63±0.28 | 0.73±0.17 | 0.96±0.18 | 0.87±0.15 | 0.32±0.28 | 0.20±0.18 | 0.26±0.25 | 222(32.8%) |
|  | P value | **<0.001** | **<0.001** | 0.675 | **<0.001** | 0.896 | 0.902 | 0.331 | 677 |
| Spherical 19 | Consistency | 0.79±0.22 | 0.92±0.10 | 0.93±0.10 | 0.92±0.09 | 0.30±0.23 | 0.21±0.17 | 0.38±0.31 | 201(43.0%) |
|  | Inconsistency | 0.67±0.33 | 0.80±0.24 | 0.90±0.13 | 0.87±0.12 | 0.28±0.23 | 0.22±0.20 | 0.33±0.29 | 266(57%) |
|  | P value | **<0.001** | **<0.001** | **<0.001** | **<0.001** | 0.343 | 0.734 | 0.074 | 467 |
| Oval  19 | Consistency | 0.68±0.32 | 0.87±0.20 | 0.85±0.13 | 0.86±0.12 | 0.27±0.19 | 0.20±0.18 | 0.20±0.21 | 328(70.2%) |
|  | Inconsistency | 0.93±0.42 | 1.08±0.19 | 0.86±0.12 | 0.93±0.10 | 0.27±0.20 | 0.20±0.18 | 0.34±0.34 | 139(29.8%) |
|  | P value | **<0.001** | **<0.001** | 0.297 | **<0.001** | 0.929 | 0.808 | **<0.001** | 467 |
| Total 66 | Consistency | 0.78±0.33 | 0.91±0.18 | 0.91±0.16 | 0.90±0.13 | 0.30±0.25 | 0.20±0.17 | 0.26±0.26 | 984(61.1%) |
|  | Inconsistency | 0.71±0.36 | 0.84±0.25 | 0.91±0.15 | 0.88±0.13 | 0.29±0.24 | 0.21±0.19 | 0.31±0.29 | 627(38.9%) |
|  | P value | **<0.001** | **<0.001** | 0.361 | **0.004** | 0.527 | 0.838 | **<0.001** | 1611 |

Bold indicates the significant difference (*P* <0.05).
